# Distribution of erosions in hands and feet at the time for the diagnosis of RA and during 8-year follow-up

**DOI:** 10.1007/s10067-020-05465-x

**Published:** 2020-10-23

**Authors:** Maria L. Andersson, B. Svensson, K. Forslind

**Affiliations:** 1 Faculty of Medicine, Department of Clinical Sciences, Rheumatology, Lund University, Lund, Sweden; 2 Spenshult Research and Development Centre, Halmstad, Sweden

**Keywords:** Disease activity, Distribution of erosions, No erosions, Radiography, Rheumatoid arthritis

## Abstract

**Background:**

Joint destruction in rheumatoid arthritis (RA) is usually evaluated by radiographs of both hands and feet, while the inflammatory status mostly is evaluated by DAS28 which, however, does not include the feet.

**Objectives:**

To investigate the distribution of erosions in hands and feet in early RA over 8 years and its potential clinical implications. Furthermore, the group of patients never showing erosions has been addressed.

**Methods:**

This study comprises 1041 patients from the BARFOT study of patients with early RA. Radiographs of hands and feet were performed at baseline, 1, 2, 5, and 8 years and evaluated by the Sharp van der Heijde scoring (SHS) method (32 joints in the hands and 12 in the feet). Disease activity was measured by DAS28, SR, CRP, and function with HAQ.

**Results:**

In the feet, there were significantly more eroded joints in percent of examined joints than in the hands at all time points. Patients with erosions only in the feet were younger, more often seropositive and smokers. They had significantly lower baseline DAS28, than the patients with erosions only in the hands. The patients without erosions over time were, at diagnosis, significantly younger and less frequently seropositive compared with patients having erosions.

**Conclusions:**

This study highlights the importance of evaluating the feet in patients with RA, both with clinical examinations and with imaging and lends support to the notion that seropositivity and smoking are risk factors for erosive disease. Further studies of patients with nonerosive disease are needed.

**Table:** 

**Key Points:** *• Foot problems are common in RA* *• This study emphasizes the limitations of DAS28 and Sharp van der Heijde score as regards evaluating disease activity and radiographic damage* *• This study highlights the importance of evaluating the feet in patients with RA with clinical examinations and imaging* *• This study also points out the need of further studies of patients with non-erosive RA.*

**Supplementary Information:**

The online version contains supplementary material available at 10.1007/s10067-020-05465-x.

## Introduction

Rheumatoid arthritis (RA) is a systemic, chronic, and progressive inflammatory disease, characterized by joint swelling, tenderness, and destruction of synovial joints and eventually irreversible loss of physical function [1]. It has since long been recognized that presence of bone erosions is a hallmark of RA, driven by the inflammatory process resulting in various degrees of joint destruction and disability.

The distribution of erosions at the diagnosis of RA may vary and are most commonly detected in the hands and/or feet. Accordingly, the methods for scoring erosions are based on conventional radiographs of hands and feet.

Involvement of the feet is very common in early RA patients [2], with the forefoot (metatarsophalangeal joints) usually being the first anatomical location where the symptoms are noticed [3–5]. At diagnosis, up to 50% of patients present some kind of foot problems [6] and continuing foot involvement in patients with longstanding RA has been estimated to 30–90% [2, 7, 8].

In a number of patients, no erosions are detected at diagnosis. However, in a few cases, erosions are never detected, not even after several years. Whether this is consistent with the concept of RA does not seem to be fully established.

The main goal of the management of RA is to suppress the disease activity in order to reach a state of remission [9]. However, clinical remission criteria may overlook important aspects of RA, especially subclinical inflammatory activity and continued progression of radiographic joint damage [10, 11].

Since long, the 28 joint Disease Activity Score (DAS28), a well validated composite index, is widely used to assess disease activity in RA [12, 13]. However, a potential drawback is that this measure does not include the feet.

To our knowledge, the distribution of erosions and its possible clinical implications have previously not been addressed. Therefore, we have performed a study on 1041 patients from the BARFOT early RA cohort to explore this issue. The aims were to investigate the distribution of erosions in hands and feet in early RA over 8 years, to elucidate if the distribution might have clinical implications, e.g., on the interpretation of DAS28 in patients with a predominance of feet erosions. Furthermore, the group of patients never showing erosions, neither at diagnosis nor at follow-up, has been addressed.

## Material and methods

### Patients

In all, 2857 patients were included in the BARFOT (Better Anti-Rheumatic FarmacOTherapy) study from 1992 to 2006. Patients were ≥ 18 years of age, fulfilling the classification criteria for RA established by the American Rheumatism Association [14] and had a disease duration of ≤ 12 months. The 1041 patients who had radiographs at diagnosis with available separate data of hands and feet and at least two of four follow-ups during 8 years were included in this study. At 8 years, 842 patients participated in the follow-up; 705 of these had available radiographs.

The patients were assessed according to a structured protocol at baseline, 3 and 6 months, and at 1, 2, 5, and 8 years. The patients were treated with DMARDs in accordance with the recommended treatment strategy in Sweden as earlier described [15, 16].

### Clinical disease assessments

Disease activity was assessed by the composite index Disease Activity Score calculated on 28 joints (DAS28; range 0–9.4, best to worse) [12], C-reactive protein (CRP), and the erythrocyte sedimentation rate (ESR; 0–150 mm/h), analyzed by the Westergren method [17]. DAS28 includes the number of swollen joints (range 0–28), number of tender joints (range 0–28), and patient’s global assessment of disease activity (PatGA) measured on a visual analogue scale (VAS). Pain was assessed by a VAS (ranged 0–100 mm, best to worse).

Physician´s global assessment of disease activity (PhAss) was assessed by a 5 graded Likert scale, ranging from no disease activity to high disease activity.

Rheumatoid factor (RF) was measured according to the current laboratory standards at the participating hospitals. Antibodies to cyclic citrullinated peptides (antiCCP) were detected using the ELISA CCP2 test (Euro-Diagnostica, Malmö, Sweden).

The Swedish version of the Stanford Health Assessment Questionnaire (HAQ) was used to measure daily life function (range 0–3, best to worse) [18].

### Radiographic assessments

Posterior–anterior radiographs of the hands and feet were assessed at baseline and at 1, 2, 5, and 8 years according to the van der Heijde modification of the Sharp score (SHS) where 32 joints in the hands and 12 in the feet are assessed [19], calculating total SHS (range 0–448), erosion score (ES) (range 0–280), and joint space narrowing score (JSN) (range 0–168). Erosive disease was defined as presence of erosions on radiographs of the hands (hands and wrists) and feet at baseline, erosion score ≥ 1. Eroded joints were also assessed in percent of examined joints and percent of maximum erosion score. The films were read by one of two experienced readers. Double readings of a fraction of films showed good agreement between the two readers. The intraclass correlation coefficient for SHS was excellent (0.940–0.998).

### Statistics

Statistical analyses were performed using SPSS version 21.0 statistical software (IBM SPSS). To test the differences between groups, the independent samples *T* test was used for continuous variables, and the chi-square test was used for proportions. When comparing more than two groups, ANOVA post hoc (Tukey) analyses were performed. All significance tests were 2-tailed and conducted at the 0.05 level of significance.

## Results

### Demographic and clinical characteristics

Demographic and clinical characteristics for all 1041 patients at diagnosis are shown in Table 1. The mean age was 56 years and 68% were women, 68% were seropositive (RF and/or antiCCP positive), and 27% were current smokers. The mean disease duration was 6 months, DAS28 5.06, pain VAS 45, ESR 32, HAQ 0.94, and erosion score 2.

**Table 1:** Demographic, clinical and radiographic characteristics of all patients, and divided into four groups, at diagnosis and 8 years

	Baseline						8-year follow-up					
	All	No erosions	Erosions in hands	Erosions in feet	Erosions in both hands and feet		All	No erosions	Erosions in hands	Erosions in feet	Erosions in both hands and feet	
	Mean (SD)	Mean (SD)	Mean (SD)	Mean (SD)	Mean (SD)	**p value	Mean (SD)	Mean (SD)	Mean (SD)	Mean (SD)	Mean (SD)	*p value
*N* (%)	1041	590 (57)	191 (18)	104 (10)	153 (15)		842	493	140	90	119	
Age, year	56 (15)	54 (15)	62 (14)	52 (14)	62 (12)	< 0.001						
Gender, female, %	68	69	68	72	64	0.572						
RF, % (St.R)	62	56 (− 1.7)	65 (0.6)	76 (1.7)	71 (1.4)	< 0.001						
AntiCCP, % (St.R)	53	48 (− 1.5)	50 (− 0.5)	79 (2.9)	65 (1.3)	< 0.001						
Seropos., % (St.R)	68	62 (− 1.6)	69 (0.2)	85 (2.1)	75 (1.1)	< 0.001						
Never smoker, % (St.R)	43	45 (0.7)	41 (− 0.5)	32 (-1.7)	46 (0.5)							
Current smoker, % (St.R)	27	25 (− 0.9)	28 (0.3)	47 (3.8)	20 (− 1.7)	< 0.001						
Prev. smoker, % (St.R)	30	30 (0)	31 (0.3)	21 (-1.5)	34 (1.0)							
Duration, months	6 (3.1)	6 (3.1)	6 (3.2)	6 (3.3)	6 (3.1)	0.631						
DAS28	5.06 (1.28)	5.07 (1.31)	5.18 (1.26)	4.71 (1.34)	5.11 (1.14)	0.024	2.93(1.31)	2.87 (1.31)	2.98 (1.33)	3.09 (1.34)	3.00 (1.32)	0.485
PhAss												
No, % (St.Res)	2	2 (0.2)	2 (0.1)	0 (-1.2)	2 (0.5)		43	42	49	35	45	
Low, % (St.Res)	22	23 (0.4)	19 (− 1.0)	35 (2.7)	15 (− 1.8)	0.032	45	46	41	49	39	0.186
Moderate, % St.Res)	56	56 (0.2)	56 (0.1)	45 (-1.4)	60 (0.7)		10	9	9	13	16	
High, % (St.Res)	22	19 (-0.8)	23 (0.9)	20 (− 0.1)	23 (0.7)		2	2	1	3	0	
PatGA	45 (25.5)	46 (25.9)	44 (25.8)	41 (25.4)	43 (23.8)	0.162	29.9 (25.8)	30.0 (25.8)	32.1 (27.4)	31.8 (27.2)	25.4 (22.1)	0.175
ESR	32 (24.4)	29 (22.8)	38 (26.0)	33 (26.0)	39 (25.0)	< 0.001	17.5 (16.0)	15.5 (14.0)	20.6 (19.4)	15.5 (13.3)	23.4 (19.0)	< 0.001
CRP	30 (35.4)	28 (35.6)	34 (36.7)	28 (30.7)	33 (35.1)	0.104	8.6 (14.8)	7.7 (9.7)	8.8 (11.7)	7.8 (6.9)	12.9 (31.0)	0.007
Pain	45 (24.5)	47 (24.2)	43 (24.7)	43 (24.5)	42 (25.2)	0.034	29.8 (25.1)	29.8 (25.3)	32.4 (26.5)	29.3 (25.0)	26.7 (22.9)	0.359
HAQ	0.94 (0.63)	0.96 (0.64)	0.97 (0.63)	0.90 (0.66)	0.86 (0.53)	0.291	0.61 (0.62)	0.60 (0.59)	0.70 (0.72)	0.65 (0.65)	0.54 (0.56)	0.216
ES	2 (3.8)	0	3 (2.5)	3 (2.7)	8 (5.6)	< 0.001	5.6 (5.6)	5.2 (5.1)	6.5 (6.2)	6.2 (6.2)	5.8 (6.1)	0.098
JSN	4 (7.4)	1 (3.2)	7 (8.3)	5 (6.9)	11 (10.7)	< 0.001	8.0 (10.9)	4.1 (6.9)	11.4 (11.0)	13.1 (17.2)	16.2 (10.9)	< 0.001
SHS	6 (9.9)	1 (3.3)	10 (9.3)	8 (7.9)	19 (13.6)	< 0.001	17.3 (17.9)	10.3 (13.6)	24.3 (16.2)	24.6 (21.5)	33.0 (17.4)	< 0.001

*RF* rheumatoid factor, *St.R* standardized residual, *antiCCP* anticyclic citrullinated peptides, *Seropos.* RF and/or antiCCP positive, *DAS28* disease activity score of 28 joints, *PhAss* physician´s global assessment of disease activity, *PatGA* patient global assessment, *ESR* erythrocyte sedimentation rate, *CRP* C-reactive protein, *HAQ* health assessment questioner, *SOFI* signal of functional impairment, *ES* erosion score, *JSN* joint space narrowing, *SHS* Sharp van der Heijde score

After 8 years, 199 patients were lost to follow-up, of whom 58% were women. These patients were at diagnosis older, had higher mean ESR and HAQ as well as radiographic scores.

The clinical characteristics of the 842 patients, who completed the 8-year follow-up, are shown in Table 1. The mean DAS28 was 2.93, pain VAS 30, ESR 17, HAQ 0.61, and erosion score 8.

### Distribution of erosions in hands and/or feet

The patients were divided into four groups according to the distribution of erosions at diagnosis—no erosions (57%), erosions only in the hands (18%), erosions only in the feet (10%), and erosions in both hands and feet (15%).

At diagnosis, significant differences were found between the groups regarding age, seropositivity, current smoking, DAS28, PhAss, ESR, pain VAS, SHS, ES and JSN but not regarding gender, disease duration, PatGA, CRP, or HAQ (Table 1).

**Fig. 1:**
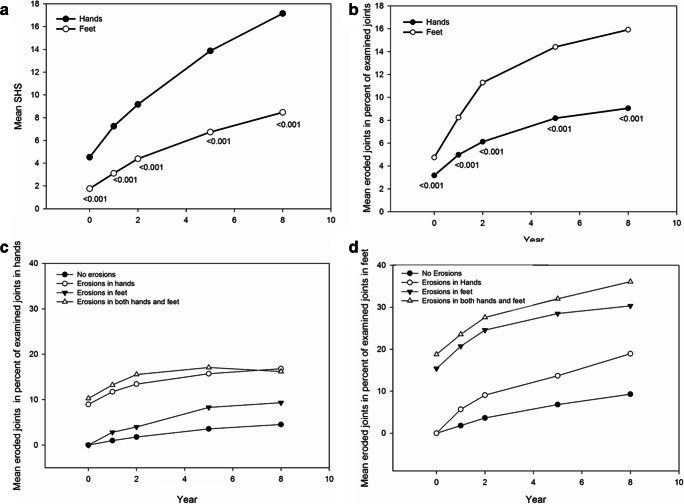
Mean Sharp van der Heijde score (SHS) (**a**), eroded joints in percent of examined joints in hands and feet during 8 years (**b**) and eroded joints in percent of examined joints in hands (**c**) and feet (**d**) in the groups divided by erosions at diagnosis during 8 years

The mean SHS was consistently higher in the hands (Fig. 1a) than in the feet but when the mean number of eroded joints was calculated in percent of examined joints, the feet had significantly more erosions than the hands at all time points during the observation years (Fig. 1b), and the feet also had higher erosion score in percent of maximum erosion score at 8 years (data not shown). Figure 1c and d shows the distribution of erosions in percent of examined joints over 8 years in hands and feet in the different groups.

Comparisons between the groups at diagnosis revealed significant differences between the groups regarding some demographic and clinical characteristics (Table 2). These were of particular interest in the groups “feet only” and “no erosions” and will be described separately.

**Table 2:** Post hoc comparisons between the groups of patients with different distribution of erosions at diagnosis and at 8 years

	Group 1	Group 2	Group 3	Group 4	ANOVA	Tukey post hoc analysis
	No erosions mean (SD)	Hands only mean (SD)	Feet only mean (SD)	Hands and feet mean (SD)	*p* value	Groups significantly different
Age, years	54 (15)	62 (14)	52 (14)	62 (12)	< 0.001	1 + 2; 1 + 4; 2 + 3; 3 + 4
DAS28 (0)	5.07 (1.31)	5.18 (1.26)	4.71 (1.34)	5.11 (1.14)	0.024	1 + 3, 2 + 3
ESR(0)	29 (22.8)	38 (26.0)	33 (26.0)	39 (25.0)	< 0.001	1 + 2; 1 + 4;
Pain (0)	48 (24.2)	44 (24.7)	44 (24.5)	42 (25.2)	0.034	
ES (0)	0	3 (2.5)	3 (2.7)	8 (5.6)	< 0.001	1 + 2; 1 + 3; 1 + 4; 2 + 4; 3 + 4
JSN (0)	1 (3.2)	7 (8.3)	5 (6.9)	11 (10.7)	< 0.001	1 + 2; 1 + 3; 1 + 4; 2 + 3; 2 + 4; 3 + 4
SHS (0)	1 (3.3)	10 (9.3)	8 (7.9)	19 (13.6)	< 0.001	1 + 2; 1 + 3; 1 + 4; 2 + 3; 2 + 4; 3 + 4
ESR 8 years	15 (14.0)	21 (19.4)	15 (13.3)	23 (19.0)	< 0.001	1 + 2, 1 + 4, 3 + 4
CRP 8 years	8 (9.7)	9 (11.7)	8 (6.9)	13 (31.0)	0.007	1 + 4
ES 8 years	4 (7)	11 (11)	13 (17)	16 (11)	< 0.001	1 + 2, 1 + 3, 1 + 4, 2 + 4, 3 + 4
JSN 8 years	10 (14)	24 (16)	25 (21)	33 (17)	< 0.001	1 + 2, 1 + 3, 1 + 4, 2 + 4, 3 + 4
SHS 8 years	14 (18)	36 (24)	38 (36)	49 (24)	< 0.001	1 + 2, 1 + 3, 1 + 4, 2 + 4, 3 + 4

*DAS28* disease activity score of 28 joints, *ESR* erythrocyte sedimentation rate, *ES* erosion score, *JSN* joint space narrowing, *SHS* Sharp van der Heijde score

There were no significant differences between the groups as to the presence of reported arthritic symptoms from large joints at diagnosis (data not shown).

The groups did not differ significantly as to treatment given at diagnosis, not regarding cDMARDs, bDMARDs, or corticosteroids (*p* = 0.37).

**Fig. 2:**
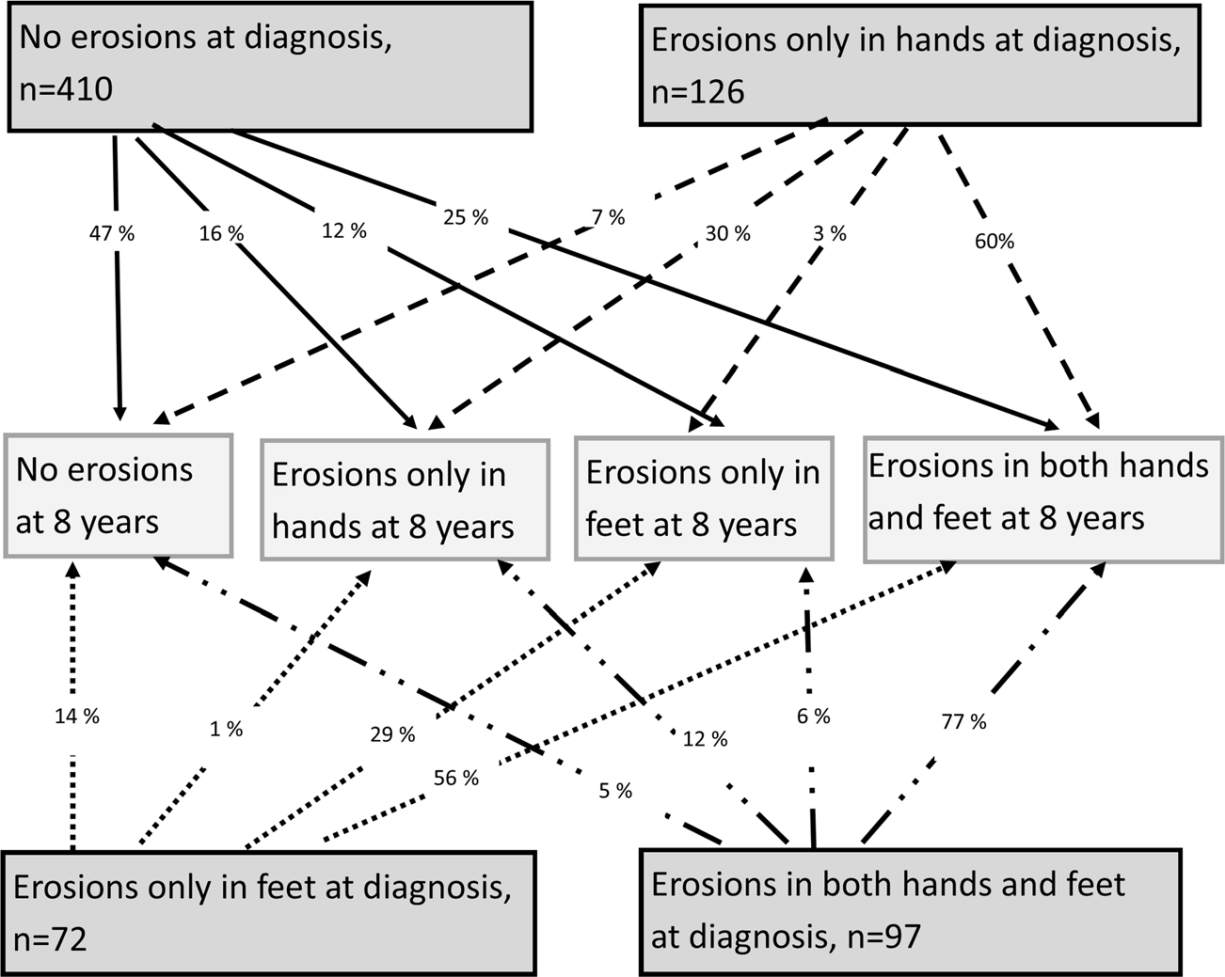
The distribution of erosions in hands and feet at diagnosis and at 8 years in the 705 patients with radiographs on both occasions

The distribution of erosions after 8 years is shown in Fig. 2. Of the patients with no erosions at diagnosis, 47% still had no erosions after 8 years. More than half of the patients with erosions in hands or feet only had erosions in both hands and feet while about one-third of them did not change location after 8 years.

The clinical (845 patients) and radiological (705 patients) outcomes at the 8-year follow-up visit in the four erosions distribution groups are shown in Table 1 with significant differences between the groups only regarding ESR, CRP, ES, JSN, and SHS.

A post hoc analysis (Table 2) revealed that the group with erosions in both hands and feet had significantly higher ESR than the no erosions and feet only groups and had also significantly higher CRP than the no erosion group.

The no erosions group had significantly lower radiographic scores than the other groups. There were no significant differences between the groups regarding DAS28, PatGA, pain, or HAQ.

Furthermore, no significant differences were noted between the groups regarding frequency of remission or persistent disease activity (data not shown).

There were no group differences over the 8 years in treatment with cDMARDs, bDMARDs, or corticosteroids (CS) (data not shown).

Repair of erosions is likely to have occurred in patients with erosions at diagnosis, who showed no erosions after 8 years, and in some cases, an erosion might have disappeared while another was new, in another location. This occurred in at least 47 patients (Fig. 2)

### Patients with erosions only in the feet

The mean erosion score at diagnosis was 3.1 in the group with erosions in feet only vs 3.4 in the group with hands only, nonsignificant. However, there were significantly more eroded joints in the feet than in the hands, in percent of examined joints, mean (SD) 4.7 (10.9) vs. 3.2 (6.1) (Fig. 1b).

During the disease course up to 8 years, the mean number of eroded joints in percent of examined joints was consistently and significantly higher in the feet only group than in the hands only group (Fig. 1c and d).

At diagnosis, the patients with erosions only in the feet were, compared with those with erosions only in the hands, significantly younger (52 vs 62 years), were more frequently smokers (47 vs 28%), and more often seropositive (85 vs 69%).

The feet only group had the lowest value for DAS28. The mean DAS28 differed significantly between the groups with different locations of the erosions at diagnosis (*p* = 0.024). In the post hoc tests, the feet-only group differed significantly from the group with no erosions (*p* = 0.048) and that with erosions only in the hands (*p* = 0.015) (Tables 1 and 2). There was no statistically significant difference between the group with erosions only in the feet and the group with erosions in both hands and feet (*p* = 0.074). Except for these significant differences in DAS28 between groups at diagnosis, no significant differences in DAS28 were seen during the disease course or after 8 years.

After 8 years, the groups differed in levels of ESR, CRP, and radiographic scores (Table 1). The post hoc analysis revealed that the feet only group had a lower mean ESR and CRP similarly to that in the no erosions group (Table 2). The hands and feet erosion group had significantly more erosions than the feet only and hands only groups.

The treatment with DMARDs or CS in the feet only group did not differ from that in the other groups, neither at diagnosis nor during the disease course or after 8 years (data not shown).

### Patients who never had any erosions compared with those who had erosions occasionally or consistently

Of the 1041 patients, 545 had radiographs performed on all five occasions and were studied to compare the patients who never had erosions with those who had erosions occasionally or consistently during the 8-year follow-up. These 545 patients were significantly younger, had a lower ESR and HAQ, fewer erosions and lower SHS at diagnosis compared with the patients lacking radiographs at one or more of the predetermined follow-up visits (data not shown).

The 545 patients were divided into two groups, the never erosive group (*n* = 138) and the group, who showed erosions on some or all assessments, the ever erosive group (*n* = 407).

At diagnosis, some significant differences between the groups were noted. Thus, compared with the ever erosive group, the patients in the never erosive group were significantly younger, were less frequently seropositive, and had more tender joints but lower ESR and CRP (Table 3).

**Table 3:** Demographic and clinical differences at diagnosis and 8 years between patients without erosions (never erosive) and patients with erosions occasionally or consistently (ever erosive)

	At diagnosis			At 8 years		
	Never erosive mean (SD)	Ever erosive mean (SD)	*p* value	Never erosive mean (SD)	Ever erosive mean (SD)	*p* value
*N* (%)	138 (25)	407 (75)		138 (25)	407 (75)	
Age, year	52 (14.5)	55 (13.2)	0.014			
Gender, female, %	67	71	0.337			
RF, %	44	69	< 0.001			
AntiCCP, %	29	63	< 0.001			
Seropos, %	53	74	< 0.001			
Never smoker, %	45	43				
Smoker, %	21	28	0.212			
Prev. smoker, %	34	29				
Duration, months	6 (3.2)	6 (3.0)	0.211			
DAS28	4.92 (1.37)	5.01 (1.27)	0.468	2.86 (1.32)	2.91 (1.26)	0.739
PhAss						
No, %	3	1		50	42	
Low, %	24	23	0.386	43	46	0.354
Moderate, %	58	58		7	10	
High, %	15	18		1	2	
SJC	9 (5.6)	9 (5.8)	0.228	1 (2.7)	2 (3.2)	0.010
TJC	8 (6.9)	7 (5.8)	0.031	3 (4.6)	2 (3.4)	0.013
PatGA	45 (26.4)	45 (25.7)	0.836	30 (25.9)	29 (25.0)	0.804
ESR	26 (20.8)	32 (23.0)	0.004	14 (15.1)	18 (15.1)	0.039
CRP	23 (27.6)	31 (35.2)	0.013	6 (6.4)	9 (18.7)	0.102
Pain	45 (25.2)	46 (24.6)	0.522	30 (25.0)	30 (24.0)	0.659
HAQ	0.90 (0.66)	0.91 (0.59)	0.929	0.59 (0.55)	0.57 (0.59)	0.695
ES	0	2 (3.9)	< 0.001	0	10 (10.5)	< 0.001
JSN	0 (1.6)	5 (7.9)	< 0.001	3 (5.2)	22 (18.2)	< 0.001
SHS	0 (1.5)	7 (10.5)	< 0.001	3 (5.2)	32 (26.1)	< 0.001
Treatment						
No DMARD, % (Std.Res.)	26 (1.7)	18 (− 1.0)		34 (2.8)	19 (− 1.6)	
cDMARD, % (Std.Res.)	57 (− 1.5)	71 (0.9)	0.008	53 (− 1.2)	63 (0.7)	< 0.001
bDMARD, % (Std.Res.)	0	0		5 (− 2.1)	13 (1.3)	
CS no DMARD, % (Std.Res.)	17 (1.5)	11 (− 0.9)		8 (1.3)	5 (− 0.8)	

*RF* rheumatoid factor, *AntiCCP* anticyclic citrullinated peptides, *Seropos*. RF and/or antiCCP positive, *DAS28* disease activity score of 28 joints, *PhAss* physician´s global assessment of disease activity, *SJC* swollen joint count, *TJC* tender joint count, *PatGA* patient global assessment, *ESR* erythrocyte sedimentation rate, *CRP* C-reactive protein, *HAQ* health assessment questioner, *ES* erosion score, *JSN* joint space narrowing, *SHS* Sharp van der Heijde score, *DMARD* disease modifying antirheumatic drugs, *c* conventional, *b* biological, *CS* corticosteroids

After 8 years, the clinical differences between the never and ever erosive groups were similar to those at diagnosis. Thus, ESR was still significantly lower, the tender joint count higher and now the swollen joint count was lower in the never erosive group (Supplement figure 1). At diagnosis, the radiological scores were significantly lower in the never erosive group (Table 3).

No significant differences were noted between the never and ever erosive groups regarding frequency of remission or persistent disease activity (data not shown).

There were distinct differences in DMARD treatment between the groups (Table 3). Thus, bDMARDs and cDMARDs were invariably less frequently given to patients who never showed erosions. In contrast, prednisolone was used in fairly similar proportions to patients in the two groups.

### Patients who never had any erosions compared with those with no erosions at diagnosis but erosions later during the disease course

Of the 545 patients with radiographs performed on all five occasions, 328 had no erosions at diagnosis, 138 continued without erosions during the disease course (never erosive group) while 190 developed erosions later (later erosive group) during the 8-year period.

At diagnosis, the never erosive group was significantly less frequently seropositive, but otherwise, the two groups were clinically very similar while SHS and JSN were significantly higher in the later erosive group (Table 4).

**Table 4:** Demographic and clinical differences at diagnosis and 8 years between patients without erosions (never erosive) and patients with no erosions at diagnosis but erosions at 1 year and/or later (later erosive)

	At diagnosis			At 8 years		
	Never erosive mean (SD)	Later erosive mean (SD)	*p* value	Never erosive mean (SD)	Later erosive mean (SD	*p* value
*N* (%)	138 (42)	190 (58)		138 (42)	190 (58)	
Age, year	52 (14)	52 (12)	0.982			
Gender, female, %	67	74	0.168			
RF, %	44	71	< 0.001			
AntiCCP, %	29	68	< 0.001			
Seropos., %	53	76	< 0.001			
Never smoker, %	45	43				
Smoker, %	21	29	0.214			
Previous smoker, %	34	28				
Duration, months	7 (3)	6 (3)	0.187			
DAS28	4.92 (1.37)	5.04 (1.24)	0.405	2.86 (1.32)	2.95 (1.32)	0.572
PhAss						
No, %	3	1		50	38	
Low, %	24	23	0.543	43	48	0.087
Moderate, %	58	61		7	12	
High, %	15	14		1	3	
SJC	9 (6)	9 (6)	0.681	1 (3)	2 (3)	0.005
TJC	9 (7)	8 (6)	0.421	3 (5)	2 (4)	0.137
PatGA	45 (26)	47 (25)	0.549	30 (26)	30 (25)	0.832
ESR	26 (21)	29 (21)	0.223	14 (15)	16 (13)	0.311
CRP	23 (28)	29 (25)	0.137	6 (6)	8 (12)	0.066
Pain	45 (25)	50 (23)	0.071	30 (25)	30 (24)	0.927
HAQ	0.90 (0.66)	0.98 (0.60)	0.304	0.59 (0.55)	0.55 (0.55)	0.472
ES	0.	0	0.241	0	7 (8)	< 0.001
JSN	0 (2)	1 (3)	< 0.001	3 (5)	15 (15)	< 0.001
SHS	0 (2)	1 (3)	< 0.001	3 (5)	22 (20)	< 0.001
*Treatment*						
No DMARD, % (Std.Res.)	26 (1.1)	18 (− 1.0)		34 (2.4)	17 (− 2.1)	
cDMARD, % (Std.Res.)	57 (− 1.2)	71 (1.0)	0.033	53 (− 1.2)	66 (1.0)	< 0.001
bDMARD, % (Std.Res.)	0	0		5 (− 1.8)	13 (1.5)	
CS no DMARD, % (Std.Res.)	17 (1.2)	11 (− 1.0)		8 (1.2)	4 (− 1.0)	

*RF* rheumatoid factor, *AntiCCP*; anticyclic citrullinated peptides, *Seropos.* RF and/or antiCCP positive, *DAS28* disease activity score of 28 joints, *PhAss* physician´s global assessment of disease activity, *SJC* swollen joint count, *TJC* tender joint count, *PatGA* patient global assessment, *ESR* erythrocyte sedimentation rate, *CRP* C-reactive protein, *HAQ* health assessment questioner, *ES* erosion score, *JSN* joint space narrowing, *SHS* Sharp van der Heijde score, *DMARD* disease modifying antirheumatic drugs, *c* conventional, *b* biological, *CS* corticosteroids

After 8 years, the groups were clinically very similar although the later erosive group had significantly more swollen joints (Supplement figure 2). This group also showed significantly higher radiographic scores (Table 4).

No significant differences were noted between the never and later erosive groups regarding frequency of remission or persistent disease activity (data not shown).

There were significant overall differences between the never and later erosive groups regarding DMARD and CS treatment at diagnosis and 8 years (Table 4). The treatment showed similar patterns at all follow-ups (data not shown). The post hoc analysis revealed that the patients in the never erosive group were less frequently treated with DMARDs compared with the patients in the later erosive group. The treatment with CS only was not significantly different between the groups.

## Discussion

This longitudinal study aimed to investigate the radiographic distribution of erosions in hands and feet in patients with early RA followed for 8 years. Fifty-seven percent of the patients had no erosions in hands or feet at baseline, and 47% of these were still erosion-free after 8 years. At baseline 18% of the patients had erosions in the hands only, 15% in both hands and feet and 10% only in the feet. There was no difference in disease duration between the groups.

The Sharp van der Heijde score (SHS) for evaluation of radiographic damage in hands and feet in patients with RA has an overweight for the hands as more joints in the hands than in the feet are included in the score [20]. Thus, SHS does not catch present damage in the feet. However, in this study, we calculated the development of erosions in percent of examined joints and found throughout the study more erosions and progression in the feet than in the hands. This is in agreement with previous studies reporting more erosions in the feet than in the hands, and also that erosions developed earlier in the feet [20–22].

van der Heijde et al. [20] reported in a study of 90 patients that at study start more foot than hand joints were affected, and that this predominance was still present after 3 years. Furthermore, Plant et al. [22] followed 114 patients for 8 years and found that the feet showed the greatest initial radiological progression. Hulsman et al. [21] in a study on 502 patients, reported that feet joints, especially the fifth metatarsophalangeal joint, generally became eroded earlier than hand joints.

At diagnosis, 25% of the patients in this study had erosions in the feet, 10% of these only in the feet. These patients were younger, more frequently smokers and had more often RF- and/or antiCCP antibodies compared with the other groups. Seropositivity and smoking are well known to be associated with high disease activity, assessed by DAS28 [23]. However, in this study, the group with erosions only in the feet had lower DAS28, despite a high rate of smokers and seropositivity. One explanation to this could be that since DAS28 does not include examination of the feet the disease activity may be underestimated in patients with inflammation mainly localized to the feet.

These observations are in agreement with those in a study by Bakker et al. [24]. They studied 265 RA patients over 5 years and found, in agreement with us, that patients with more radiographic progression in the feet were younger and more often RF-positive [24]. In that study, the patients were divided into different groups according to radiographic progression rate. They found that the patients developing radiographic progression predominantly in the feet showed, in contrast to the patients in the other groups, no corresponding change in DAS28. This was interpreted to be due to an underestimation of the disease activity measured by DAS28 among foot progressors.

These studies thus suggest that erosions at diagnosis, limited to the feet, may be associated with low values for DAS28 due to the fact that inflammation in the foot joints is not reflected by DAS28. This may be more than a marginal problem since the prevalence of baseline involvement of the feet in early RA is common and may cause undertreatment due to misleadingly low values for DAS28. In addition, patients categorized as being in remission by DAS28 have been found to have inflammation in the feet in up to 40% [8, 25], further highlighting the importance of also examining the feet. We agree with the statement of Hulsmans et al. [21] and Bakker et al. [24] that radiographs of the feet should be included in assessments of radiologic damage in clinical trials as well as in daily practice.

The patients, who never developed erosions during follow-up, were younger, less often antiCCP or RF positive and had lower ESR over time than the patients with erosions, always or at times. In addition, the never erosive group received significantly less treatment with DMARDs while corticosteroids were similarly given. Taken together, these observations suggest that the patients who never developed erosions had a milder disease, possibly different from RA. Our results are in agreement with those of Liao et al. [26], who investigated clinical predictors of erosion-free status in rheumatoid arthritis where the patients were stratified by disease duration and followed for 2 years. They found that 56 (21%) of 271 patients were still erosion-free after 2 years. The mean disease duration at study start was 3.4 years for the erosion-free patients and 4.5 years for the erosive group. The erosion-free patients were younger and less often RF- and/or antiCCP positive.

In the present study, 57% of the patients had no erosions at diagnosis, and after 8 years, 47% of these still had no erosions. In other studies, 40 to 50% of the patients were nonerosive at diagnosis, and about 4–30% remained nonerosive during follow-up periods for 2 to 10 years [20, 22, 27].

The higher percent of erosion-free patients at follow-up in our study might be due to shorter disease duration at diagnosis and improved treatment as the referred studies all were carried out in the 1980s and early 1990s.

In a cross-sectional study by Amaya-Amaya et al. [28], 110 out of 500 patients with RA were erosive after a follow-up period of median 10 years. Of these, 40 patients with a disease duration of more than 5 years were studied by plain radiographs, ultrasound (US), and computed tomography (CT). Of these only 8 (20%) were nonerosive by all three methods (by radiography 53%, US 43%, CT 50%). Accordingly, they drew the conclusion that nonerosive RA is very rare. They also performed a systematic literature search for studies evaluating nonerosive RA and associated factors. Seventeen studies reporting highly diverging prevalence were retrieved, which may be explained by the great differences between studies in disease duration, (3 months to 16 years), follow-up time (1–12 years) and study design. Factors associated with nonerosions included seronegativity and younger age, which is in agreement with the results in the present study.

Absence of erosions at study end in patients with erosions at diagnosis occurred in 24 patients and may indicate repair. Whether erosion repair occurs or not in RA has earlier been debated, but today this feature has been demonstrated in several studies with different prevalence depending on imaging modality and definition of repair [29].

Foot problems have a negative impact on the quality of life in patients with RA [25, 30]. As clinically detectable inflammation precedes erosions in the joints [31, 32], it is of importance to integrate the feet in the clinical evaluation. The importance of earliest possible antiinflammatory treatment of patients with newly diagnosed RA is well recognized [33, 34], and rapid attainment of remission can prevent or limit joint damage and maintain good quality of life. In order to achieve this, it is important recognize the presence of inflammation also in the feet.

To our knowledge, this is the first study on radiographic damage in RA, in which patients who never had erosions (never erosive) are compared both with patients who occasionally or consistently had erosions (ever erosive) and with those who developed erosions during follow-up (later erosive).

The main differences between the never erosive and the other groups was that the never erosive group was less frequent seropositive and received more seldom treatment with DMARDs. However, the mean DAS28 over time was similar in the groups, which may be explained by the observation that the never erosive group still had higher tender joint count compared with the ever and later erosions groups, significant only for the ever erosive group. These observations suggest that patients who never developed erosions may represent a milder disease than those with erosions.

In previous reports, there have been divergent reports regarding associations between radiographic damage and disability measured by HAQ.[35, 36]. In a 5-year study of 191 patients with early RA, Combe et al. [35] found radiological progression in half of the patients whereas HAQ disability improved in most of them, Ödegård et al. [36], on the contrary, found in a 10-year study that radiographic damage contributed to impaired physical function as did Andersson et al. [37] in a study of 1938 patients. Of interest, Maillefert et al. [38] followed 135 patients for 5 years and found changes in joint damage to be related to subsequent HAQ-disability due to changes in joint narrowing rather than in erosion score. In the present study, there was no significant group difference in physical function assessed by HAQ, during the 8 years.

The data presented here indicate that routine monitoring not only of radiologic damage but also of swelling and tenderness in the feet is necessary for an adequate evaluation of the disease activity.

A strength of this study is the large number of patients from a well-controlled cohort of early RA patients followed prospectively long-term with a structured protocol including radiographs.

A weakness is that, as this is a post-hoc study, we could not separate the joint counts for swollen and tender joints in the wrists and hands from the 28 joint count included in DAS28.

### Summary and conclusions

Joint destruction over time was found to be more pronounced in the feet than in the hands, predominantly in younger patients with a negative test for RF and/or antiCCP and who were smokers. Erosions at diagnosis, limited to the feet, were associated with low disease activity by DAS28 at baseline, due to the fact that inflammation limited to the feet is not reflected by DAS28. These observations have relevance for the evaluation of disease activity and joint damage progression and for treatment decisions. Inclusion of the feet in a score like DAS28 would conceivably improve the validity of this established disease activity measure.

This study highlights the importance of evaluating the feet in patients with RA, both with clinical examinations and imaging and lends support to the notion that seropositivity and smoking are risk factors for erosive disease. Further studies of patients with no erosive disease are needed—do these patients have RA or a different disease?

## Electronic supplementary material

Supplement figure 1. Panel A to E show DAS28 with the included variables (swollen and tender joint count, ESR and global health) over eight years in the patients who never had any erosions compared with those who had erosions on some occasion. (PNG 5618 kb)

High resolution image (TIF 1647 kb)

Supplement figure 2. Panel A to E show DAS28 with the included variables (swollen and tender joint count, ESR and global health) over eight years in patients who never had any erosions compared with those who had erosions on later occasions. (PNG 5452 kb)

High resolution image (TIF 1578 kb)

## Data Availability

The data analyzed during the current study are available from the corresponding author on reasonable request.
