# Supplementary figures and images for: Distribution of erosions in hands and feet at the time for the diagnosis of RA and during 8-year follow-up
Source: Clin Rheumatol. 2020 Oct 23;40(5):1799–810. doi: 10.1007/s10067-020-05465-x (PMC8102449; doi:10.1007/s10067-020-05465-x)

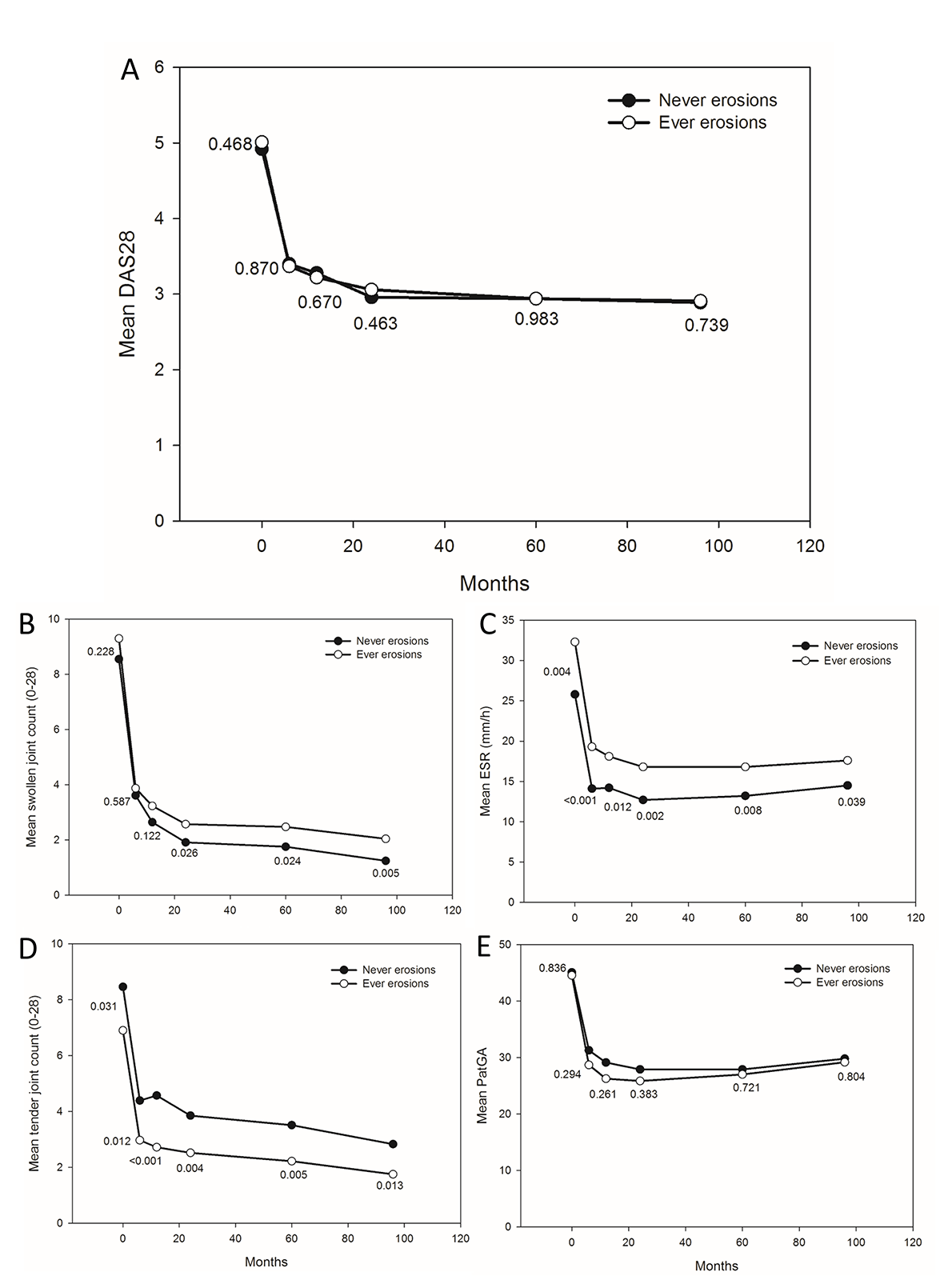

Supplement: Supplementary file 1 — Panel A to E show DAS28 with the included variables (swollen and tender joint count, ESR and global health) over eight years in the patients who never had any erosions compared with those who had erosions on some occasion. (PNG 5618 kb) [file 10067_2020_5465_Fig3_ESM.png]

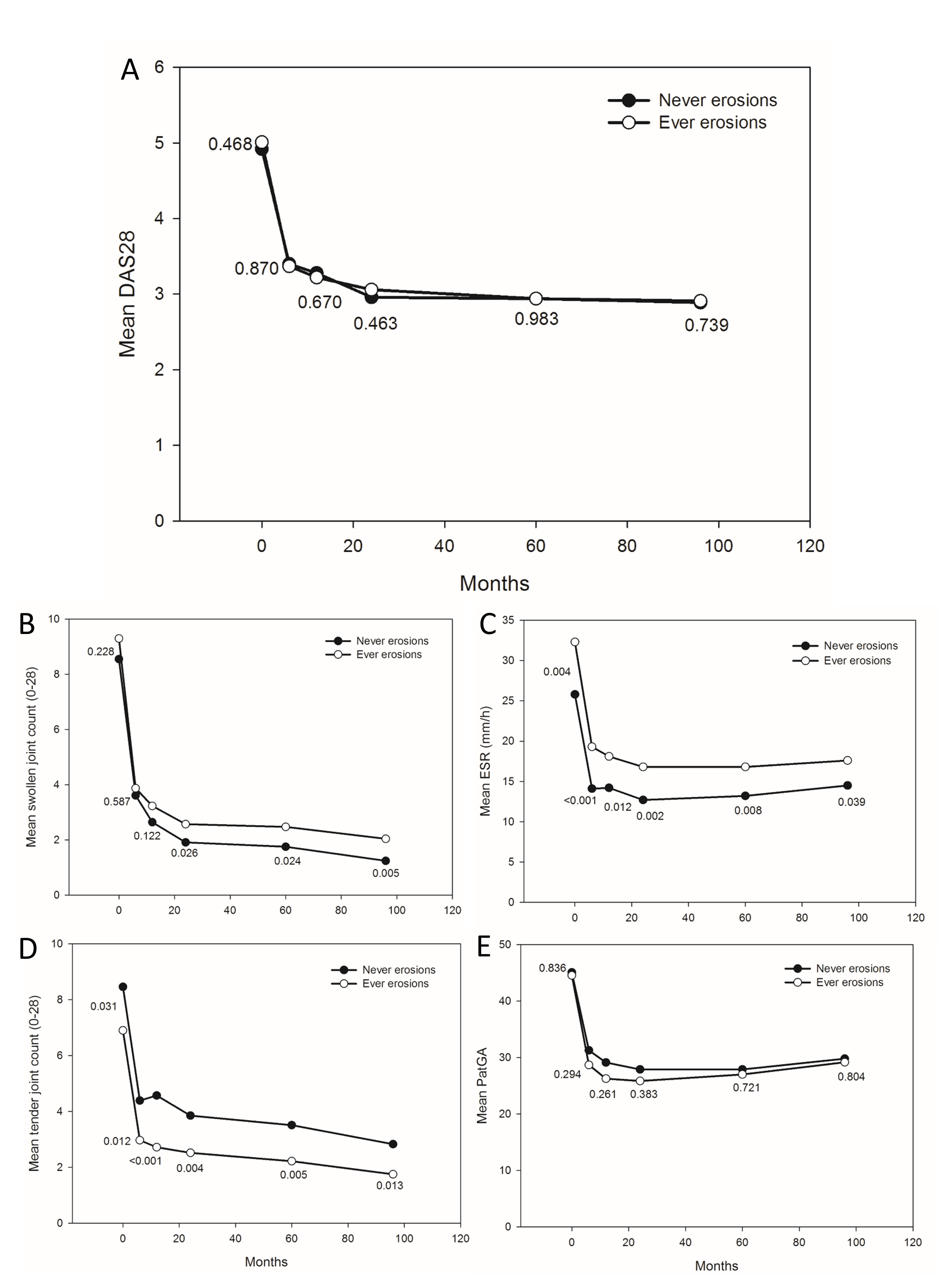

Supplement: Supplementary file 2 — High resolution image (TIF 1647 kb) [file 10067_2020_5465_MOESM1_ESM.tif]

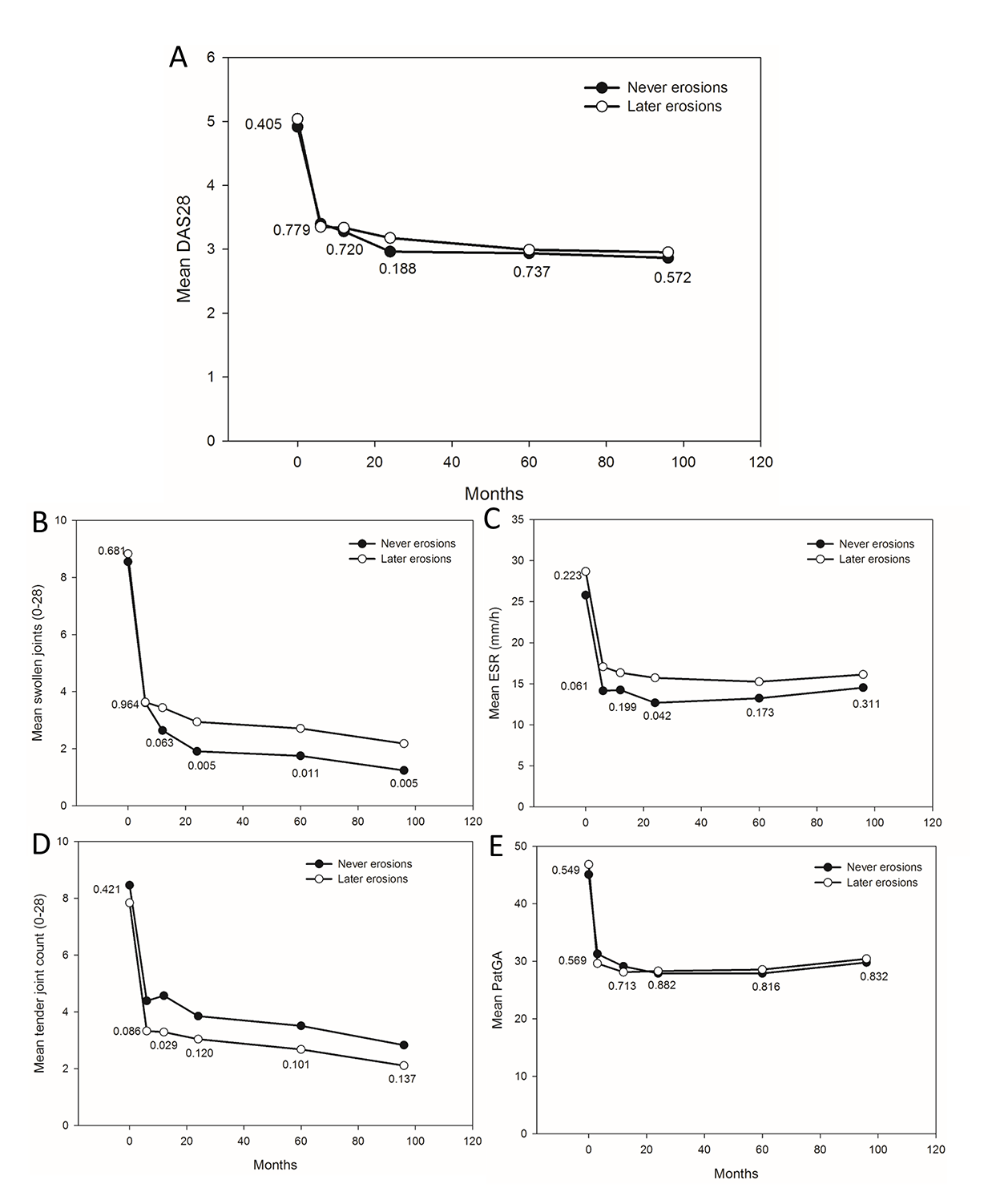

Supplement: Supplementary file 3 — Panel A to E show DAS28 with the included variables (swollen and tender joint count, ESR and global health) over eight years in patients who never had any erosions compared with those who had erosions on later occasions. (PNG 5452 kb) [file 10067_2020_5465_Fig4_ESM.png]

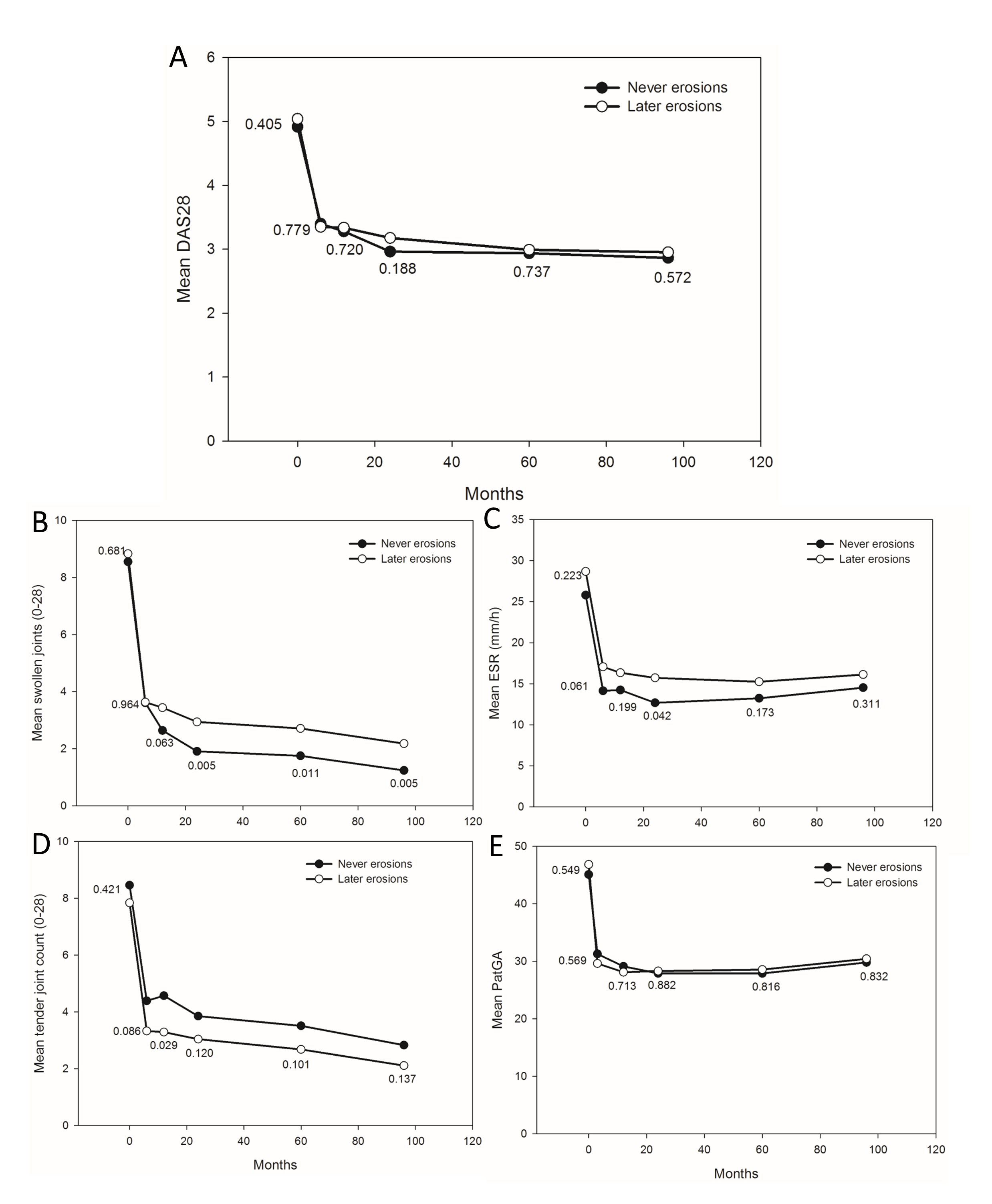

Supplement: Supplementary file 4 — High resolution image (TIF 1578 kb) [file 10067_2020_5465_MOESM2_ESM.tif]
